# A Silent Corticotroph Pituitary Carcinoma: Lessons From an Exceptional Case Report

**DOI:** 10.3389/fendo.2021.784889

**Published:** 2021-12-21

**Authors:** Pablo Remón-Ruiz, Eva Venegas-Moreno, Elena Dios-Fuentes, Juan Manuel Canelo Moreno, Ignacio Fernandez Peña, Miriam Alonso Garcia, Miguel Angel Japón-Rodriguez, Florinda Roldán, Elena Fajardo, Ariel Kaen, Eugenio Cardenas Ruiz-Valdepeñas, David Cano, Alfonso Soto-Moreno

**Affiliations:** ^1^ Unidad de Gestión Clínica de Endocrinología y Nutrición, Instituto de Biomedicina de Sevilla (IBiS), Virgen del Rocio University Hospital/Centro Superior de Investigaciones Científicas (CSIC)/University of Seville, Seville, Spain; ^2^ Unidad de Gestión Clínica de Endocrinología y Nutrición, Virgen de Valme University Hospital, Seville, Spain; ^3^ Unidad de Gestión Clínica de Oncología médica, Oncología Radioterápica y Radiofísica Hospitalaria, Virgen del Rocio University Hospital, Seville, Spain; ^4^ Unidad de Gestión Clínica de Anatomía Patológica, Virgen del Rocio University Hospital, Seville, Spain; ^5^ Unidad de Gestión de Radiodiagnóstico, Virgen del Rocio University Hospital, Seville, Spain; ^6^ Unidad de Gestión Clínica de Neurocirugía, Virgen del Rocío University Hospital, Seville, Spain

**Keywords:** pituitary tumor, silent corticothoph tumor, pituitary carcinoma, radiotherapy, temozolomide

## Abstract

Nowadays, neither imaging nor pathology evaluation can accurately predict the aggressiveness or treatment resistance of pituitary tumors at diagnosis. However, histological examination can provide useful information that might alert clinicians about the nature of pituitary tumors. Here, we describe our experience with a silent corticothoph tumor with unusual pathology, aggressive local invasion and metastatic dissemination during follow-up. We present a 61-year-old man with third cranial nerve palsy at presentation due to invasive pituitary tumor. Subtotal surgical approach was performed with a diagnosis of silent corticotroph tumor but with unusual histological features (nuclear atypia, frequent multinucleation and mitotic figures, and Ki-67 labeling index up to 70%). After a rapid regrowth, a second surgical intervention achieved successful debulking. Temozolomide treatment followed by stereotactic fractionated radiotherapy associated with temozolomide successfully managed the primary tumor. However, sacral metastasis showed up 6 months after radiotherapy treatment. Due to aggressive distant behavior, a carboplatine-etoposide scheme was decided but the patient died of urinary sepsis 31 months after the first symptoms. Our case report shows how the presentation of a pituitary tumor with aggressive features should raise a suspicion of malignancy and the need of follow up by multidisciplinary team with experience in its management. Metastases may occur even if the primary tumor is well controlled.

## Introduction

Most pituitary tumors are benign although they often invade surrounding structures. However, pituitary carcinomas are extremely rare accounting for only 0.1-0.2% of all pituitary tumors. This prevalence may be likely underestimated due to the challenge in diagnosing pituitary carcinoma (1). As defined by the World Health Organization, pituitary carcinomas are tumors of pituitary origin that have cerebrospinal and/or systemic metastases. Importantly, there are no histological features that can help to discriminate between pituitary carcinoma and non-metastasic pituitary tumors (2).

Literature has changed the landscape of aggressive, atypical pituitary tumors and pituitary carcinomas. The 2017 WHO classification of tumors of the pituitary gland removed the term atypical and set a number of histopathological characteristics with a high probability of recurrence (such as elevated proliferative activity or some subtypes of pituitary tumors) without a defined cut-off for Ki-67, mitotic count or p53 immunoreactivity. Thus, the description and definition of risk characteristics are highly relevant but without a separate denomination. Raverot et al. argued for a clinicopathological classification that includes aggressiveness and invasiveness, with aggressive and invasive tumors being more prone to metastatic dissemination (up to 10% of these cases) (3).

Pituitary carcinomas carry a poor prognosis with a median survival time of 1-2.6 years depending on whether systemic metastasis is found. A recent study has reported overall survival rates for pituitary carcinoma of 57.1% and 28.6% at 1 and 5 years, respectively (4). Treatment of pituitary carcinoma usually involves a multimodal therapy approach combining surgery, radiation and medical therapy. However, due to their rarity, there is a lack of reliable information to establish a standard treatment (3).

Here, we describe the multidisciplinary team experience in managing the only case of pituitary carcinoma diagnosed in our reference center over the span of 12 years. We discuss the diagnosis and treatment of this patient and review the recent literature about pituitary carcinoma.

## Case Description

**Figure 1:**
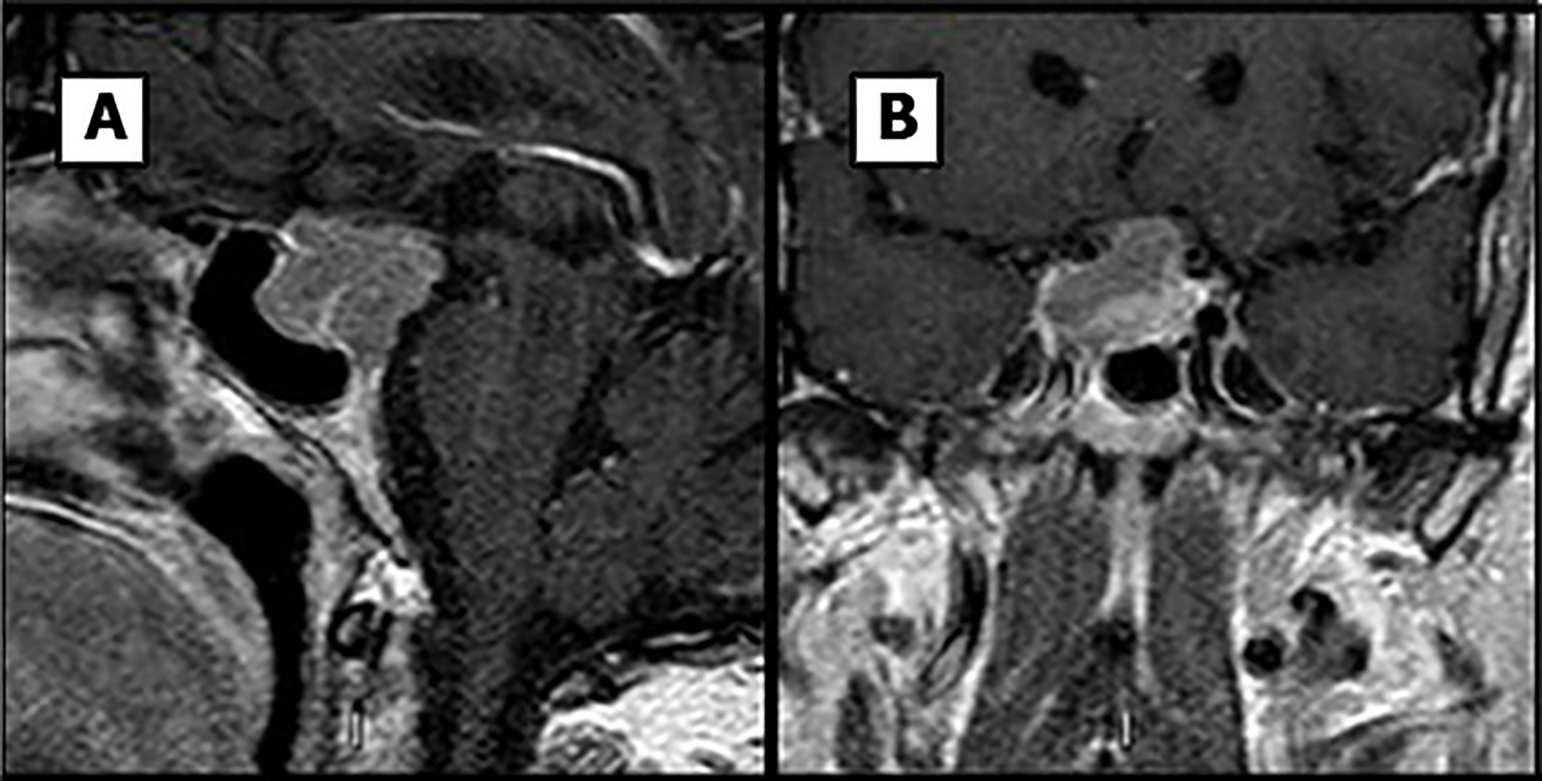
MR images previous to first surgery. Sagital **(A)** and coronal **(B)** T1-weighted images.

A 61-year-old man presented in the emergency room in 2015 complaining of headache. Physical examination revealed third-cranial nerve palsy on the left side and bitemporal hemianopsia. The patient had a history of hypertension, type 2 Diabetes Mellitus and referred loss of libido. No signs or symptoms of hormone hyperproduction were noticed, Cushing disease was ruled out by clinical signs but not by other laboratory testing such as free urinary cortisol or suppression test due to scarce pretest clinical probability. There was not relevant family history. Computed tomography imaging revealed a sellar mass with erosion of the sphenoid bone and invasion into the cavernous sinus regions and extension into the hypothalamus. A subsequent magnetic resonance imaging (MRI) scan of the brain region confirmed a 25-mm pituitary mass lesion extending into both left and right cavernous sinuses (Knosp grade 3) (**Figures 1A, B**). This lesion showed isointensity and hyperintensity on T1- and T2-weighted MRI, respectively. Laboratory test results revealed central hypogonadism and hypothyroidism and prolactin levels of 292 µUI/mL.

**Figure 2:**
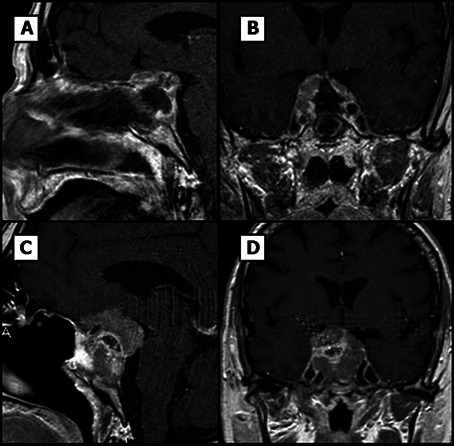
Pituitary MR images. **(A, B)** Sagital and coronal T1-weighted postsurgical images. **(C, D)** Sagital and coronal T1-weighted images, tumor rest growing can be noticed from small capsular rest to 20 mm invasive tumor.

Endoscopic endonasal transsphenoidal surgery was performed and subtotal resection was achieved. Postoperative MRI (48 hours after surgery) revealed a tumor remnant (20x7x20 mm) (**Figures 2A, B**). The patient developed permanent postoperative diabetes insipidus but third-cranial nerve palsy improved and was discharged on hormone replacement therapy with oral desmopresine (0.1 mg twice a day), hydrocortisone (20 mg/day divided three times a day) and levothyroxine (50 mcg once a day). Hydrocortisone was initiated after hypotension and hypoglycemia after surgery, hormonal determination after surgery is shown in **Table 1**.

**Table 1:** Hormonal laboratory determination 24 hours after first pituitary surgery.

Cortisol*	307 nmol/L*
FSH	0,9 UI/L
LH	0,4 UI/L
Testosterone	0,1 nmol/L
Prolactin	4 µUI/mL
GH	<0,05 ng/mL
IGF1	149 ng/mL

*Determination of cortisol after iniciation of high dose of hydrocortisone due to clinical signs of hypocorticism.

**Figure 3:**
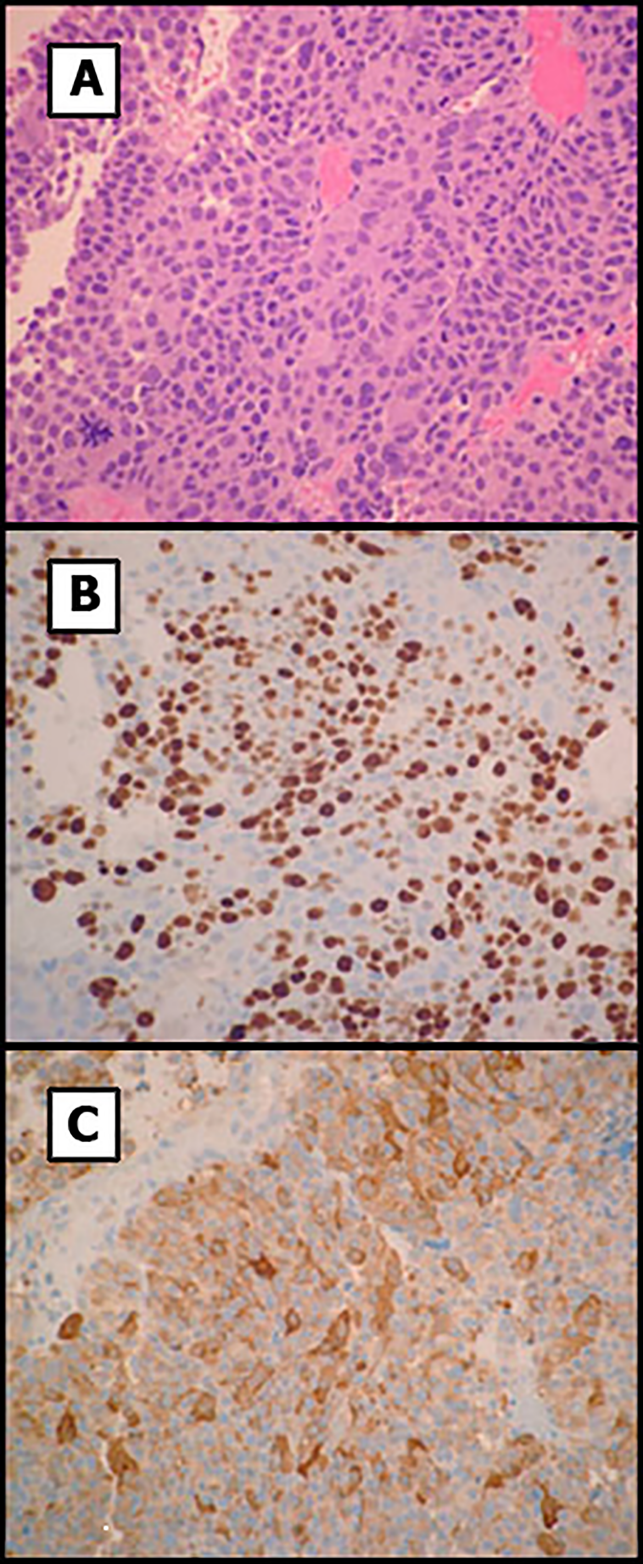
Histology of the pituitary tumor. **(A)** Primary tumor showed solid and papillary proliferation of basophilic cells with increased nuclear atypia and mitoses. **(B)** Ki-67 labeling showed areas with elevated proliferation index. **(C)** Tumor cells were positive for ACTH. Original magnification x200.

Two months after surgery, the patient presented in the emergency room with acute headache, worsening of third-cranial nerve palsy and visual loss. Computed tomography imaging surprisingly showed regrowth of the pituitary mass with a maximum diameter of 36 mm, erosion of the sphenoid bone and invasion of the sinus (**Figures 2C, D**). Histological examination of the previous surgery showed a solid and papillary neoplasm composed of slightly basophilic cells (**Figures 3A-C**). Nuclear atypia, multinucleation and mitotic figures were frequent (bigger than 2/10 HPF but not specifically quantified in the report). These cells were positive for ACTH and negative for other pituitary hormones. Ki-67 labeling index was 50% overall but higher (50-70%) in many areas of the tumor, a feature that was alerted in the pathology report. Thus, was classified as invasive, aggressive silent corticotroph tumor with high proliferation index (Grade 2b suspected of malignancy according to Trouillas et al. classification) (5).

Based on the histological findings and rapid progression of the tumor, our multidisciplinary team decided to perform a second surgery and administer postoperative chemotherapy adjuvant treatment with Temozolomide. The patient underwent only partial resection of the tumor due to extensive invasion of the sphenoid bone. A 13-mm tumor remnant could be observed postsurgically. No operative complications were developed. Although no specific symptoms were noted, a screening for metastasis was performed prior to chemotherapy. Computed tomography (CT) imaging of thorax and abdomen and MRI of the spine revealed no metastatic lesions.

**Figure 4:**
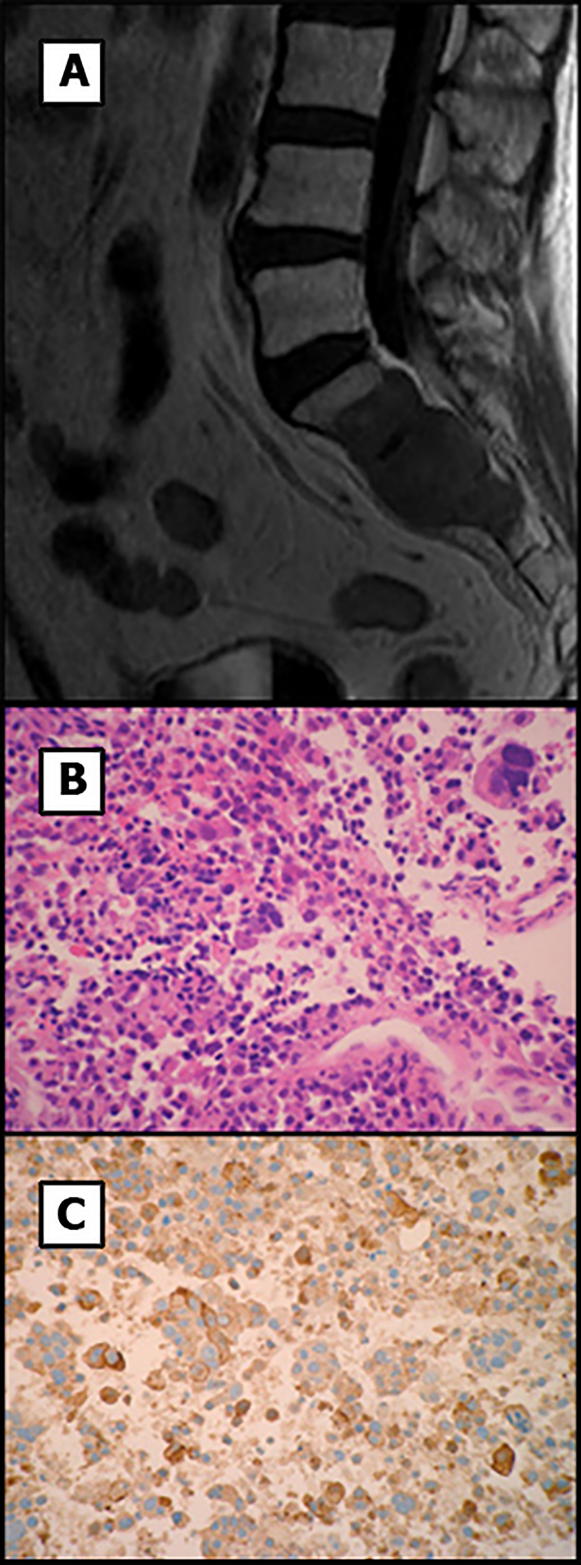
MR image and histology of the sacral mass. **(A)** T1-weighted MR image shows a highly invasive sacral metastasis. **(B)** Tumor metastasis showed solid and papillary proliferation of atypical cells including numerous giant multinucleated cells. **(C)** Tumor cells were positive for ACTH. Original magnification x200.

**Figure 5:**
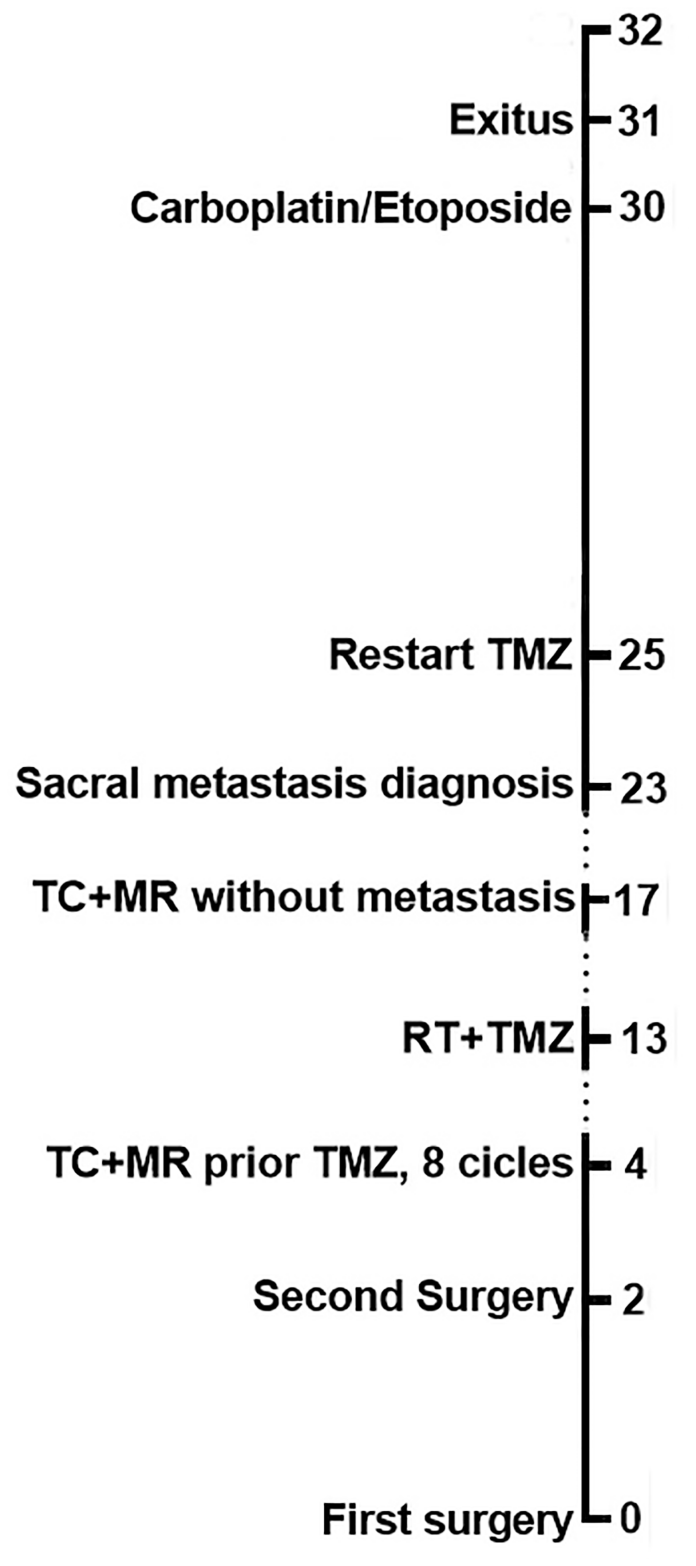
Clinical timeline of case report landmarks in months.

Temozolomide treatment was initiated 16.14 weeks after surgery at a dose of 200 mg/m^2^/day for 5 of every 28 days and was well tolerated by the patient. After four cycles, a MRI was performed and revealed size stabilization of the size of the lesion. This led our multidisciplinary team to decide to additionally treat the patient with fractionated stereotactic radiation therapy. Thus, after the eighth temozolomide cycle treatment and 13 months after the initial diagnosis, radiation therapy was administered at a total dose of 50 Gray in 28 fractions and temozolomide dose was switched to 75 mg/m^2^/day. MRI was performed three months after starting radiation therapy that did not show growth of the tumor remnant. CT of the thorax and abdomen was negative for metastatic disease. Six months after the start of the radiation therapy, the imaging tests were repeated, and the tumor lesion remained stable and campimetric improvement was also achieved. However, the CT scan now showed a mass at S1-S3 level (**Figure 4A**) compatible with metastasis. A biopsy of the sacral region was obtained. Histological examination showed a solid and papillary neoplasm positive for ACTH, consistent with metastasis of the primary pituitary tumor (**Figures 4B, C**). In this specimen nuclear atypia was pronounced with the presence of numerous giant tumor cells. Ki67 labeling index was 70-80%. Sacrectomy was not considered due to the extensive invasion of the metastatic lesion and temozolomide treatment at a dose of 200 mg/m^2^/day was restarted. Despite restarting temozolomide, the patient developed severe bilateral lower extremity weakness and the chemotherapy treatment was changed to carboplatin AUC 5 and etoposide 100 mg/m^2^ three days administered every 21 days. After one cycle of treatment the patient experienced febrile neutropenia likely due to an urinary tract infection. Due to the poor patient’s general condition, radiation therapy of the sacral region was not performed. Unfortunately, and despite aggressive antibiotic treatment, the patient died 124.4 weeks after the first surgery. A clinical timeline is shown in **Figure 5**.

## Discussion

The prevalence of incidentally found pituitary tumors in the general population is relatively high. Most of them are benign adenohypophyseal tumors commonly named as pituitary adenomas but a little unknown percentage are aggressive, invasive, resistant or even metastatic tumors. Nowadays, only proven metastatic tumors are carcinomas but a growing body of evidence show that we may have to reconsider some non-metastatic pituitary tumors as tumors with malignant potential (6). We report an additional case of pituitary carcinoma that initially presented as an aggressive pituitary tumor with a rapid metastatic dissemination despite an adequate chemotherapy treatment and radiotherapy of primary lesion. This exceptional case highlights two difficult aspects of aggressive tumors: the diagnosis of malignancy and the management of these aggressive tumors.

Despite intensive research on the subject, predicting an aggressive behavior of pituitary tumors remains an unmet clinical need. Currently, there are not clinical, radiological, surgical and pathological markers of malignancy, except the metastasis that is the reason why until now only the tumors with metastasis are considered as malignant and named “carcinoma”. However, in our patient some unusual signs may suggest the malignancy of the primary tumor which was clinically an aggressive tumor.

First of all, initial clinical symptom was third-cranial-nerve palsy which is actually a clinical manifestation of cavernous sinus invasion proven by RMI.

Secondly, aggressiveness and prompt regrowth after the first surgery. It only took two months for the tumor to grow to a similar size as before the first surgery, triggering third-cranial-nerve palsy once more.

Finally, the pathology report gives several clues about the potential inner nature of our tumor. Corticotroph tumor, especially the silent one, is the tumor type the most frequent carcinoma with the lactotroph one (7). In the 2017 WHO classification for pituitary tumors, silent corticotroph tumor is a special subtype a with high probability of recurrence and more aggressive behavior (2). All this, together with the elevated Ki-67 labeling index (≥10%) prompted us to perform screening for metastasis. The use of Ki-67 as a marker for aggressive behavior in pituitary tumors has long been suggested but no clear consensus exists on the cut-off values that can distinguish between pituitary carcinoma and benign tumors (8, 9). Nonetheless, a particularly high Ki-67 labeling index may be suggestive of malignant potential, as described in several reports (10, 11). In agreement with this notion, the observed Ki-67 labeling index in our patient was extremely high compared to most published data on pituitary tumors, and more specifically, pituitary carcinomas (11). A Ki-67 index ≥10% is found in aggressive pituitary tumors as well as pituitary carcinomas and this may be because many aggressive tumors are, in fact, malignant (6).

As is noted by Trouillas et al, although probably the vast majority of pituitary tumors will have a benign behavior, there is a need for an integrative classification which allows to identify malignant potential in order to improve clinical results with an early diagnosis of complications. Trouillas et al. proposed a five grade clinicopathological classification of pituitary endocrine tumors which included a tumor grade based on invasive and proliferative characteristics. So, invasive and proliferative tumors are classified as Grade 2b with a poor prognosis and an increased probability of progression. These tumors could be considered as suspected of malignancy as 6/8 carcinomas from the original series were classified as Grade 2b at the initial surgery and with an identification of metastasis during follow-up in 10% of the initial Grade 2b tumors (3, 5).

Current recommendations in metastasis screening refer structural and/or functional imaging studies should be considered in aggressive tumors in the evidence of discordant biochemical and radiological findings in the absence of site-specific symptoms. Due to previously analyzed tumor characteristics, we considered our pituitary tumor as suspect of malignancy (3) from the second surgery with initial metastasis screening before first cycle of temozolomide and periodical screening after that. We decided to perform thorax and abdomen CT and spine MRI rather than 18F-FDG PET/CT. Up to our knowledge, there is no enough evidence on the best study for screening of pituitary metastasis of pituitary carcinoma. 18F-FDG PET/CT could be more sensitive to small metastasis, but in our case and due to local aggressiveness we thought both studies could give similar sensitivity but with higher specificity.

The treatment of aggressive pituitary tumors and pituitary carcinoma usually combines radiation and medical therapy. First of all, clinical and hormonal presurgical analysis is mandatory in every patient with adenohypophyseal tumors found in RMI because it can lead to adjuvant medical treatment. In prolactinomas, adjuvant medical treatment with dopamine agonist could delay surgical treatment event in giant ones (12). Some subtypes of adenomas have some clinical special features because of the previously mentioned probability of progression and recurrence as lactotroph adenoma in men, sparsely granulated somatotroph adenoma or silent corticotroph adenoa. Until recently, no clear guidelines were available to help clinicians to manage aggressive and malignant pituitary tumors. The European Society of Endocrinology (ESE) published clinical practice guidelines for the management of aggressive pituitary tumors and carcinomas in 2018 (13). The authors acknowledge the limitations of these guidelines since the literature on aggressive pituitary tumors and carcinomas is scarce. However, additional support for the development of the clinical guidelines was obtained from a survey of ESE members specifically designed for that purpose (7). Transcranial or transsphenoidal surgery is the treatment of choice for patients with pituitary tumors. Surgery rarely achieves cure but it can provide relief of symptoms, particularly those associated to mass effect. Tumor recurrence in pituitary carcinoma is very common and additional therapy approaches such as radiation and chemotherapy are thus required. ESE guidelines recommend discussion within a multidisciplinary team regarding additional surgeries prior to consideration of other treatment options. In our patient, even though a diagnosis of carcinoma was not initially confirmed, the aggressive features of the pituitary tumor raised the suspicion of a malignant potential. Thus, we decided to perform an additional surgery and adjuvant chemotherapy to control tumor growth. The alkylating agent temozolomide is the most common chemotherapeutic option for aggressive pituitary tumors and carcinomas (7, 14, 15) and ESE guidelines recommend the use of temozolomide as first-line chemotherapy for aggressive pituitary tumors and carcinomas even though the evidence is based mostly on case reports and small series rather than randomized clinical trials (13). It has been difficult to obtain precise response rates to temozolomide from the literature because of heterogeneity in treatment regimens, and definitions in disease control but it is considered that about half of the patients do not respond to temozolomide treatment (15). Importantly, patients receiving concurrent temozolomide treatment and radiotherapy seem to respond more often (7). ESE guidelines suggest that temozolomide response should be assessed after three cycles of therapy and that treatment can be extended according to their tolerance and clinical response. In our patient, temozolomide treatment was well tolerated and considering that tumor size remained stable we decided to use a lower dose of temozolomide (75 mg/m^2^) concomitant with radiation therapy, as in the Stupp protocol. However, the identification of metastasis during the follow-up led us to restart temozolomide monotherapy at the highest recommended dose. Temozolomide treatment was largely ineffective though that combined with the rapid progression of the tumor forced us to consider an alternate oncological treatment. Unfortunately, the literature about second and third line treatments for temozolomide-resistant pituitary tumors is scarce and the reported chemotherapeutic agents have been very heterogeneous. Indeed, the recent ESE guidelines were unable to provide recommendations in this regard. We decided initiated chemotherapy with a combination carboplatin and etoposide [as standard treatment for certain poorly differentiated neuroendocrine tumors (16, 17)] and local radiotherapy treatment for sacral mass later, but the efficiency was limited due to severe complications developed. Indeed other treatments were foreseen, as immunotherapy, mTOR inhibitors and even peptide receptor radionuclide therapy, but according to aggressiveness, histopathological characteristics, refraction to temozolomide and requirements of aggressive treatment, a platine scheme was selected as is contemplated by Raverot et al. in ESE Clinical Practice Guidelines (11, 18). Possible scheme that could be used after the onset of sacral metastasis is capecitabine-TMZ combination (CAPTEM). This combination could enhance TMZ results due to attenuation effect of MGMT DNA repair activity by capecitabine. CAPTEM has been used in different clinical settings with good tolerance and morphological response. Better results have been described when CAPTEM has been used as first line or as second line treatment, particularly in those cases in which TMZ resistance has not been achieved. In TMZ-resistant pituitary tumors, CAPTEM can achieve a partial response in some cases but its efficacy seems to be lower (18).

In conclusion, our case illustrates how the presentation of a pituitary tumor with aggressive features should raise suspicion of malignancy. There remains a need to identify other treatment options for aggressive pituitary tumors and carcinoma.

## Data Availability Statement

The raw data supporting the conclusions of this article will be made available by the authors, without undue reservation.

## Ethics Statement

Ethical review and approval was not required for the study on human participants in accordance with the local legislation and institutional requirements. The patients/participants provided their written informed consent to participate in this study. Written informed consent was obtained from the individual(s) for the publication of any potentially identifiable images or data included in this article.

## Author Contributions

PR-R collected and analyzed the data, and wrote the report. EV-M atended the patient, ED-F atended the patient. JC made literature review. IF atended the patient. MA atended the patient. MJ-R performed the histological examination. FR interpreted MRI image. EF interpreted MRI image. AK performed surgery. ECR-V performed surgery. DC analyzed the data and wrote the report. AS-M coordinated the research, analyzed the data and wrote the report. All authors contributed to the article and approved the submitted version.

## Funding

This work was supported by grants from the ISCIII-Subdirección General de Evaluación y Fomento de la Investigación co-funded with Fondos FEDER (PI16/00175 to AS-M and DC) and the Sistema Andaluz de Salud (A-0003-2016 and A-0006-2017 to AS-M, C-0015-2014 and RC-0006-2018 to DC).

## Conflict of Interest

The authors declare that the research was conducted in the absence of any commercial or financial relationships that could be construed as a potential conflict of interest.

## Publisher’s Note

All claims expressed in this article are solely those of the authors and do not necessarily represent those of their affiliated organizations, or those of the publisher, the editors and the reviewers. Any product that may be evaluated in this article, or claim that may be made by its manufacturer, is not guaranteed or endorsed by the publisher.

## Abbreviations

MRI, Magnetic resonance imaging; ACTH, adrenocorticotropic hormone; CT, Computed tomography; ESE, European Society of Endocrinology.
